# Molecular Mechanism of Isocupressic Acid Supresses MA-10 Cell Steroidogenesis

**DOI:** 10.1155/2012/190107

**Published:** 2012-05-15

**Authors:** Kuan-Hao Tsui, Jyun-Yuan Wang, Leang-Shin Wu, Chih-Hsien Chiu

**Affiliations:** ^1^Department of Obstetrics and Gynecology, Kaohsiung Veterans General Hospital, 386 Ta-Chung 1st. Road, Kaohsiung 81346, Taiwan; ^2^Department of Obstetrics and Gynecology, National Yang-Ming University School of Medicine, Taipei, Taiwan; ^3^Laboratory of Animal Physiology, Department of Animal Science and Technology, College of Bio-Resources and Agriculture, National Taiwan University, Taipei 10673, Taiwan

## Abstract

Consumption of ponderosa pine needles causes late-term abortions in cattle and is a serious poisonous plant problem in foothill and mountain rangelands. Isocupressic acid (IA) is the component of pine needles responsible for the abortifacient effect, its abortifacient effect may be due to inhibition of steroidogenesis. To investigate the more detail molecular mechanism, we used MA-10 cell, which is wild used to investigate molecular mechanism of steroidogenesis, to characterize the molecular mechanisms underlying the actions of IA in more detail. In this report, we focus on the function of IA on important steroidogenic genes, including steroidogenic acute regulatory protein (StAR), cytochrome P450 cholesterol side-chain cleavage (P450scc), and 3*β*-hydroxysteroid dehydrogenase (3*β*-HSD). We found that IA does not affect enzyme activities of these genes but inhibits transcription of P450scc and translation of StAR and P450scc through attenuating cAMP-PKA signaling. Thus, steroid productions of cells were suppressed.

## 1. Introduction

**Figure 1:**
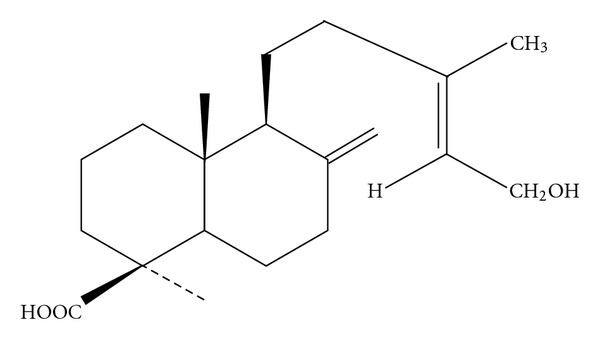
Structure of isocupressic acid.

Since the 1900s, studies have reported that ingestion of ponderosa pine needles can induce abortion in pregnant cattle; in particular, cows in late gestation have a high incidence of abortion after pine needle consumption, [1–3]. Controlled feeding trials have demonstrated the abortifacient effects of the pine needles, and this finding was confirmed by other numerous studies [4, 5]. After many attempts to identify the toxin in pine needles responsible for this effect [6], isocupressic acid (IA, Figure 1) was identified as the abortifacient compound [3, 5, 7]. Besides IA itself, arecents study has shown that agathicmetabolite of IA also induce abortion in cattle, while IA is the major abortifacient compound [8]. IA is thought to disrupt uterine vascular flow resulting in decreased placental perfusion. However, IA does not appear to act directly on the uterine artery smooth muscle, and its specific mechanism of action remains unknown. Since IA does not affect embryo implantation in cattle [9], thus, its abortifacient effects may due to inhibition of steroidogenesis.

It is well known that progesterone is required while pregnancy. Progesterone can induce development of endometrium, maintain the function of placenta, inhibit uterine contractility, and maintain the stage of pregnancy. Biosynthesis of progesterone is undergoing a process named steroidogenesis. In female animals, steroid hormones are synthesized in the corpus luteum and endometrium; in male animals, steroid hormones are synthesized in the testes. The steroid hormone testosterone, produced by Leydig cells in the testes, is involved in many important physiologic functions such as stimulating the development and growth of sex organs, promoting secondary sex characteristics, and increasing muscle mass. 

Steroidogenesis in Leydig cells is regulated by luteinizing hormone (LH). LH binds to its cognate receptors on the cell membrane, thereby activating G-protein, which in turn activates adenylyl cyclase. Activated adenylyl cyclase increases cytoplasmic cAMP concentrations [10], triggering the phosphorylation of protein kinase A (PKA) and the expression of steroidogenic genes such as steroidogenic acute regulatory protein (StAR) and cytochrome P450 cholesterol side-chain cleavage (P450scc) enzyme. StAR, P450scc, and 3*β*-hydroxysteroid dehydrogenase (3*β*-HSD) play critical roles in basal and hormone-regulated steroidogenesis [11]. StAR protein transfers free cholesterol from the cytoplasm to the mitochondrial inner membrane, where cholesterol is converted to pregnenolone by P450scc [12]. Pregnenolone is then transported to the smooth endoplasmic reticulum and converted to progesterone by 3*β*-HSD. Progesterone is converted to testosterone by further enzymatic processing.

We previously reported that IA inhibits steroidogenesis in primary bovine luteal cells [13]. However, there have been no additional reports of IA effects on steroidogenesis on male gonad cells.

Therefore, in the present study, we aimed to assess the pathologic actions of IA on reproduction by assessing its effects on steroidogenesis. The expression of steroid hormones and steroidogenic enzymes was evaluated in IA-treated MA-10 cells to characterize the molecular mechanisms underlying IA inhibition of steroidogenesis.

## 2. Material and Methods

### 2.1. Reagents and Chemicals

Cell culture medium DMEM/F12 and Medium 199, trypsin-EDTA (0.25%), penicillin G, streptomycin sulfate, fetal bovine serum (FBS), Hank's balanced salt solution, (HBSS) and trypan blue stain were purchased from Invitrogen Corporation (Grand Island, NY, USA). Collagenase type 1 was purchased from Worthington Biochemical Corporation (Lakewood, NJ, USA). Bovine serum albumin (BSA) and other general chemicals were purchased from Sigma (St. Louis, MO, USA), and 8-bromo-cyclic AMP was purchased from Tocris Bioscience (Ellisville, MO, USA). Pregnenolone and 22hydroxycholesterol (22R-OHC) were purchased from Steraloids, Inc. (Newport, RI, USA). IA was purchased from Logan Natural Products (Plano, TX, USA). 

### 2.2. Primary Mouse Leydig Cell Culture

Male C57BL/6 mice (10–12 weeks old) were purchased from National Taiwan University, maintained under 12-h light (0900-2100)/12-h dark (2100-0900) conditions and allowed free access to chow and water. All experimental protocols were approved by the Animal Care and Use Committee, College of Medicine, National Taiwan University. All procedures conformed to the National Institutes of Health Guide for the care and use of laboratory animals.

After mice were killed by decapitation, testes were removed and decapsulated in Medium 199. Then the seminiferous tubules were separated, washed once with isolation buffer (1× HBSS containing 0.1% BSA and 200 U/mL collagenase type 1), and incubated at room temperature in isolation buffer for an additional 5 min. The seminiferous tubules and cells were filtered with 250 mesh, and then the cells were collected by centrifugation at 300 ×g for 5 min and resuspended in 10 mL Medium 199. Live cells were counted with trypan blue stain, and then seeded (10^6^/mL) in culture tubes (Corning Inc, NY, USA). Cells were immediately treated with different doses of cAMP and IA and incubated at 37°C with 5% CO_2_. After 0.5, 1, or 4 h, the culture medium was collected and stored at −20°C until the enzyme immunoassay (EIA) was performed. 

### 2.3. Culture of MA-10 Cells

MA-10 mouse Leydig tumor cells were seeded into T-75 cell culture flasks (Corning Inc.) with DMEM/F-12 medium, supplemented with 10% FBS, 2.2 mg/mL sodium bicarbonate, 100 U/mL penicillin, and 0.1 mg/mL streptomycin and incubated at 37°C with 5% CO_2_. Before experiments, nonadherent cells were removed by aspiration, and healthy cells were collected by trypsinization and centrifugation. The cell pellets were gently resuspended in DMEM/F-12 medium without FBS, and cells were seeded into 48-well plates (5 × 10^4^/well), treated with different chemicals, and incubated at 37°C with 5% CO_2_. After treatment, the culture medium was collected and stored at −20°C until EIA was performed. 

### 2.4. MTT Assay

MA-10 cells cultured in 96-well culture plates (2 × 10^4^/well) plates were treated with IA in the presence or absence of cAMP. After a 4-h incubation, the cells were washed twice with DMEM/F-12 medium without FBS, and then treated with 200 *μ*L 3-(4,5-dimethylthiazol-2-yl)-2,5-diphenyltetrazolium bromide (MTT) solution for an additional 1.5 h. The MTT solution was then removed by aspiration, and MTT crystals were solubilized with 200 *μ*L dimethyl sulfoxide. The optical density (OD) of each sample was assessed at 540 nm using a dual wavelength reader (Dynatech, Denkendort, Germany). Data are present as multiple of control. 

### 2.5. Enzyme Immunoassay for Progesterone and Testosterone

The progesterone assay was modified from a direct EIA using G7, an IgM monoclonal antibody with specific affinity of 1.1 × 10^10^/M, as described previously [13–15]. G7 exhibits cross-reactivity of <0.01% with BSA and other steroids including pregnenolone, testosterone, estradiol, and estrone. Aliquots (50 *μ*L) of diluted medium and horseradish peroxide-linked progesterone conjugate (150 *μ*L) were added to microtiter plates coated with 200 *μ*L G7 (representing a 1 : 40,000 dilution). After a 15-min incubation at room temperature with gentle shaking and two washes with Tween-20 in 0.01 M phosphate buffer (pH 7.0), the color was developed with 3.7 mM O-phenylenediamine (200 *μ*L) in 0.03% H_2_O_2_ for an additional 15 min. The reaction was stopped by adding 8 N H_2_SO_4 _(50 *μ*L). The optical density (OD) of each sample was determined using a dual wavelength reader (Dynatech, Denkendort, Germany) set between 490 and 630 nm. Progesterone concentration was determined with a standard curve. Coefficients of variation were 7% (within assays) and 12% (between assays). The sensitivity of the assay was 0.3 pg/mL [14]. All standards and samples were assayed in duplicate.

### 2.6. RNA Extraction and cDNA Synthesis

Total RNA was extracted from MA-10 cells with Trizol reagent (Invitrogen) according to the manufacturer's instructions. Genomic DNA was digested with Ambion *TURBO DNA-free* DNase (Ambion, Austin, TX, USA) according to the manufacturer's instructions. To synthesize cDNA, total RNA (1 *μ*g) was mixed with 2.5 *μ*M oligo(dT) and 500 *μ*M deoxynucleotide triphosphate and denatured at 65°C for 5 min. After cooling on ice, the mixture was combined with 40 U RNaseOUT RNase inhibitor (Invitrogen), 5 mM DTT, and 200 U SuperScript III reverse transcriptase (Invitrogen) and incubated at 50°C for 60 min. The reaction was inactivated by heating to 70°C for 15 min, and the cDNA was stored at 4°C.

### 2.7. Quantitative Real-Time PCR

The quantitative real-time PCR was performed with the StepOne Real-Time PCR System (Applied Biosystems, Foster City, CA, USA) according to the manufacturer's instructions. Briefly, the 10-*μ*L reaction mixture contained cDNA, 0.5 *μ*M primer pairs, and 2× Maxima SYBR Green/ROX qPCR MasterMix (Fermentas, Burlington, Ontario, Canada). PCR amplification was carried out at 95°C for 10 min and 40 cycles of 95°C for 15 sec, 60°C for 30 sec, and 72°C for 30 sec, followed by melting curve analysis. PCR primers are shown in Table 1. 

### 2.8. Western Blot Analysis

After treatment, the cells were rinsed twice with cold phosphate-buffered saline (PBS) and harvested. Then the cells were resuspended in cold lysis buffer (2% SDS, 50 mM Tris [pH 6.8], 5 mM EDTA, 1% 2-mercaptoethanol, 5% glycerol), and whole-cell extracts were prepared, as described previously [16]. Samples containing 40 *μ*g protein were separated by 12% SDS-polyacrylamide gel electrophoresis, as described previously [16]. The separated proteins were transferred to a polyvinylidene fluoride membrane. The membrane was blocked by incubating in PBS containing 0.01% Tween-20 (PBST) and 2.5% BSA for 8 h at room temperature, followed by incubation with rabbit polyclonal primary antibodies specific for StAR [15, 17], P450scc, 3*β*-HSD [15, 18], PKA (Abcam, Cambridge, UK), total PKA (Abcam), and *β*-actin (Millipore, Temecula, CA, USA) in PBST overnight at 4°C. After four washes with PBST, the membrane was incubated for 2 h with peroxidase-conjugated goat anti-rabbit IgG (1 : 7000 dilution; Jackson ImmunoResearch, West Grove, PA, USA). The membrane was washed with PBST, and bound antibodies were visualized by the ECL system (Millipore) using Kodak X-OMAT film (Eastman Kodak Co., Rochester, NY, USA). 

### 2.9. Data Analysis

Western blot experiments were performed three times, and a representative result is shown. Densities of blots were quantified with VisionWorksLS Image Acquisition and Analysis Software (UVP, Upland, CA, USA). Results are presented as multiple of control. Results are expressed as mean ± standard error of the mean (SEM) of triplicate samples from three individual experiments. Real-time PCR and Western blot data were analyzed with Student's *t*-test using Microsoft Excel 2007. Results of other assays were analyzed by one-way ANOVA followed by Duncan's multiple comparison using SigmaStat 3.5 (Aspire Software International, Ashburn, VA, USA). *P* < 0.05 was considered significant.

## 3. Results

### 3.1. Effect of IA on Testosterone Secretion by Primary Mouse Leydig Cells

**Figure 2:**
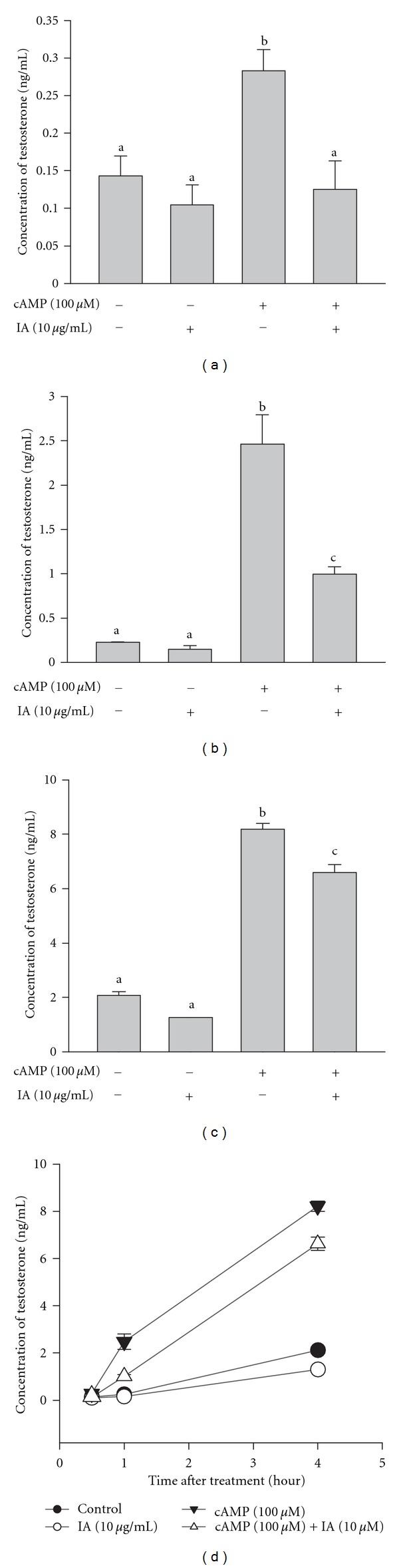
Isocupressic acid attenuates cAMP-stimulated steroidogenesis in primary mice Leydig cells. Primary mice Leydig cells were treated with or without cAMP and isocupressic acid (IA) for 0.5 h (a), 1 h (b), or 4 h (c). Results are combined in Figure 2(d). The cell culture medium was collected, and progesterone concentration was determined by EIA. Results are expressed as mean ± SEM. Each column with different letters is significantly different at *P* < 0.05.

To determine the effects of IA on steroidogenesis of the reproductive system, primary mouse Leydig cells were cultured with cAMP to stimulate testosterone production and treated with IA. As shown in Figure 2, testosterone levels of cells stimulated with cAMP (0.5, 1, or 4 h) were 2-fold to 4-fold that of controls (*P* < 0.05); however, cotreatment with IA reduced testosterone production to approximately 50% at 0.5 h, to 50% at 1 h, and to 80% at 4 h compared with cells exposed to cAMP alone (*P* < 0.05). 

### 3.2. Effect of IA on Cell Viability and Progesterone Secretion by MA-10 Cells

**Figure 3:**
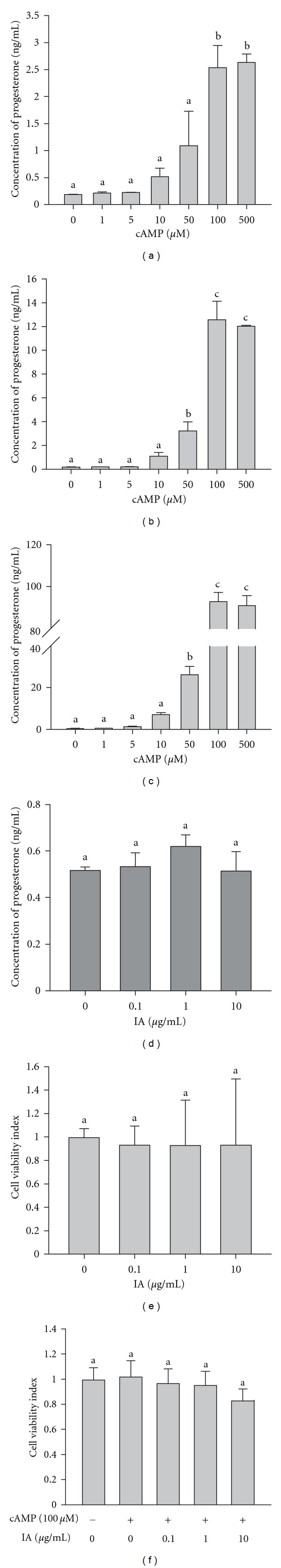
Effect of cAMP and isocupressic acid on progesterone secretion and viability of MA-10 cells. MA-10 cells were treated with increasing concentrations of cAMP for 0.5 h (a), 1 h (b), or 4 h (c), and increasing concentrations of IA for 4 h (d). The culture medium was collected, and progesterone concentration was determined by EIA. Then, MA-10 cells were treated with different doses of IA alone (e) or with 100 *μ*M cAMP (f) for 4 h. Cell viability was determined with the MTT assay. Results are expressed as mean ± SEM. Each column with different letters is significantly different at *P* < 0.05.

**Figure 4:**
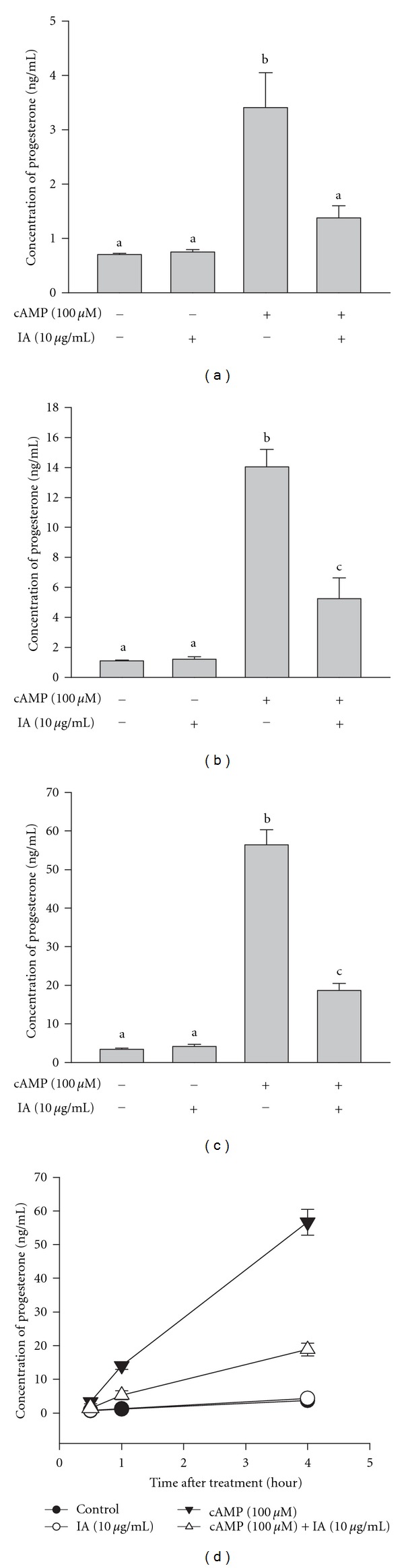
Isocupressic acid attenuates cAMP-stimulated steroidogenesis in MA-10 cells. MA-10 cells were treated with or without cAMP and IA for 0.5 h (a), 1 h (b), or 4 h (c); results are combined in Figure 4(d). The culture medium was collected, and progesterone concentration was determined by EIA. Results are expressed as mean ± SEM. Each column with different letters is significantly different at *P* < 0.05.

To determine the molecular mechanism underlying the action of IA on steroidogenesis, we used the MA-10 mouse Leydig cell line in this study. To determine the optimal cAMP concentration for our experiments, MA-10 cells were treated with cAMP (1–500 *μ*M) for 0.5, 1, or 4 h (Figures 3(a), 3(b), and 3(c)). We found that 100 *μ*M cAMP induced the most progesterone production at all time points compared to other doses of cAMP. A 4-h treatment with different doses of IA did not alter basal progesterone production in MA-10 cells (Figure 3(d)). In addition, cell viability was not affected by treatment with 10 *μ*g/mL IA alone or with cAMP (Figures 3(e) and 3(f). However, 10 *μ*g/mL IA decreased cAMP-stimulated progesterone production to about 50% that induced by 100 *μ*M cAMP alone at all time points (*P* < 0.05) (Figure 4). Further, progesterone production stimulated by 500 *μ*M cAMP was significantly decreased by 10 *μ*g/mL IA (data not shown). Taken together, these results indicate that IA inhibits cAMP-stimulated steroidogenesis without inducing cytotoxicity in MA-10 cells.

### 3.3. Effect of IA on Basal and 22R-OHC or Pregnenolone-Supported Progesterone Secretion by MA-10 Cells

**Figure 5:**
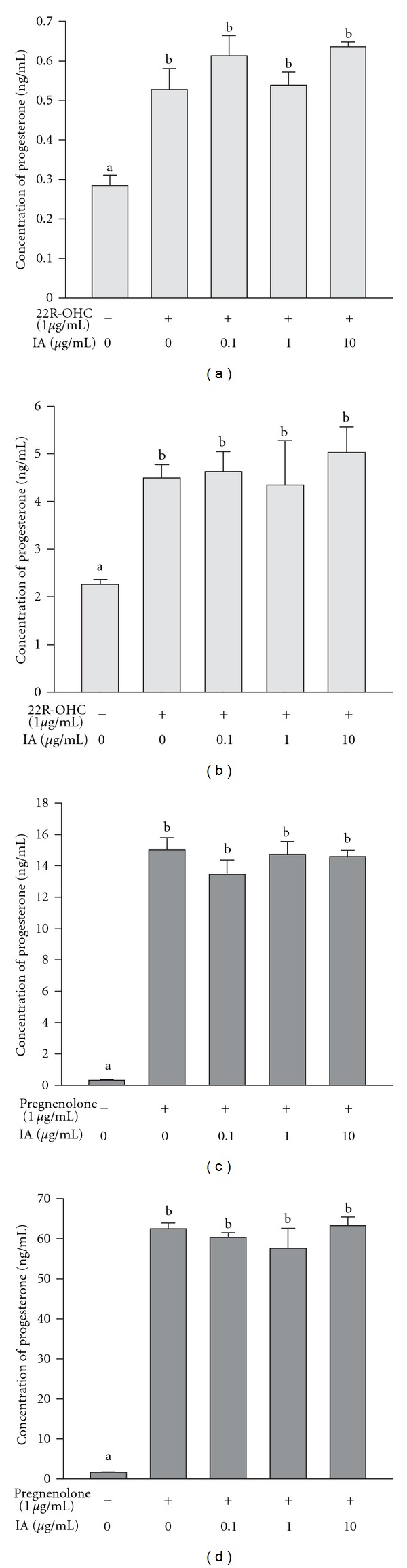
Effect of isocupressic acid on steroidogenesis in MA-10 cells incubated with 22R-OHC or pregnenolone. MA-10 cells were incubated with or without 22R-OHC (1 *μ*g/mL) and treated with different doses of IA for 0.5 h (a) or 4 h (b). MA-10 cells were also incubated with or without pregnenolone (1 *μ*g/mL) and treated different doses of IA for 0.5 h (c) or 4 h (d). The culture medium was collected, and progesterone concentration was determined by EIA. Results are expressed as mean ± SEM. Each column with different letters is significantly different at *P* < 0.05.

Because IA inhibited progesterone production of cAMP-stimulated MA-10 cells, we evaluated whether IA modulates the activity of steroidogenesis-associated enzyme, P450scc and 3*β*-HSD. MA-10 cells were treated for 0.5 or 4 h with IA (0.1, 1, or 10 *μ*g/mL) with or without 22R-OHC or pregnenolone, which are substrates in the progesterone synthesis pathway: 22R-OHC bypasses the action of StAR, and pregnenolone bypasses both StAR and P450scc. As shown in Figures 5(a) and 5(b), progesterone production was not decreased by treatment with IA and 22R-OHC for 0.5 or 4 h. Similarly, progesterone production was not decreased by treatment with IA and pregnenolone for 0.5 or 4 h (Figures 5(c) and 5(d)). Taken together, these results show that the effects of IA on steroidogenesis are not mediated by inhibiting the enzyme activity of P450scc or 3*β*-HSD in MA-10 cells.

### 3.4. Effect of IA on Steroidogenic-Associated Gene Expression in MA-10 Cells

**Figure 6:**
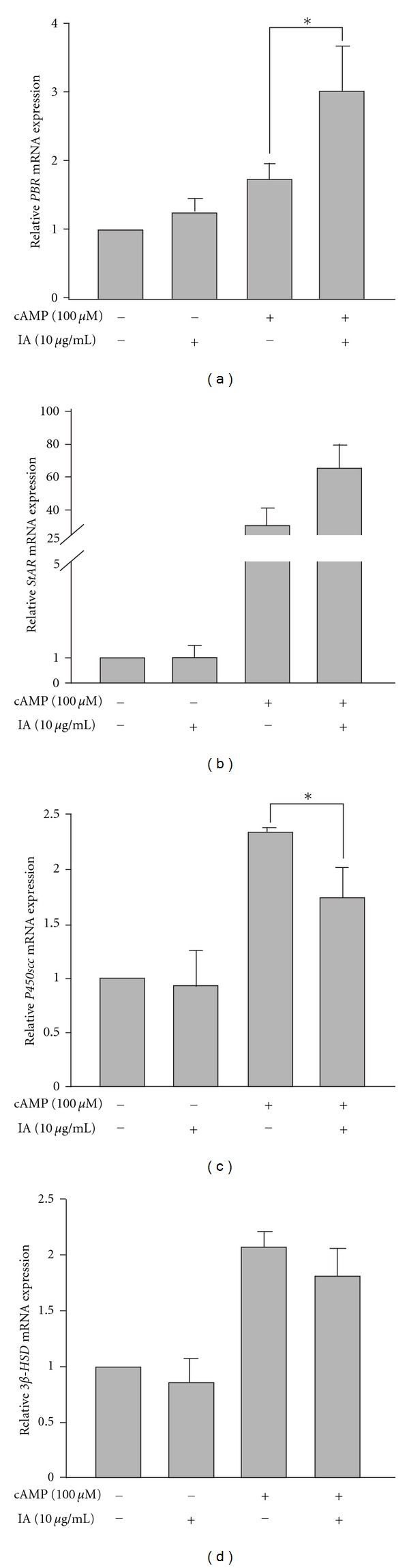
Effect of isocupressic acid on mRNA levels of steroidogenic genes in MA-10 cells. MA-10 cells were treated with or without cAMP and IA for 4 h. Expression of steroidogenic genes *PBR* (a), *StAR* (b), *P450scc* (c), and *3*β*-HSD* (d) was determined by real-time PCR. Results are expressed as mean ± SEM. The statistically significant difference between two indicated groups is labeled (*P < 0.05).

Because IA did not alter the enzyme activity of P450scc or 3*β*-HSD, we evaluated the effect of IA on the expression of the steroidogenic-associated gene including peripheral-type benzodiazepine receptor (PBR), StAR, P450scc, and 3*β*-HSD. As determined by real-time PCR (Figure 6), cAMP stimulated expression of StAR and P450scc; however, after cotreatment with IA, mRNA levels of P450scc were decreased to 70% (*P* < 0.05). The mRNA levels of PBR were significant increased after cotreatment with 10 *μ*g/mL IA (*P* < 0.05), while expression of StAR mRNA was appeared to increase but not significant (*P* = 0.06).

### 3.5. Effect of IA on Steroidogenic-Associated Protein Expression of MA-10 Cells

**Figure 7:**
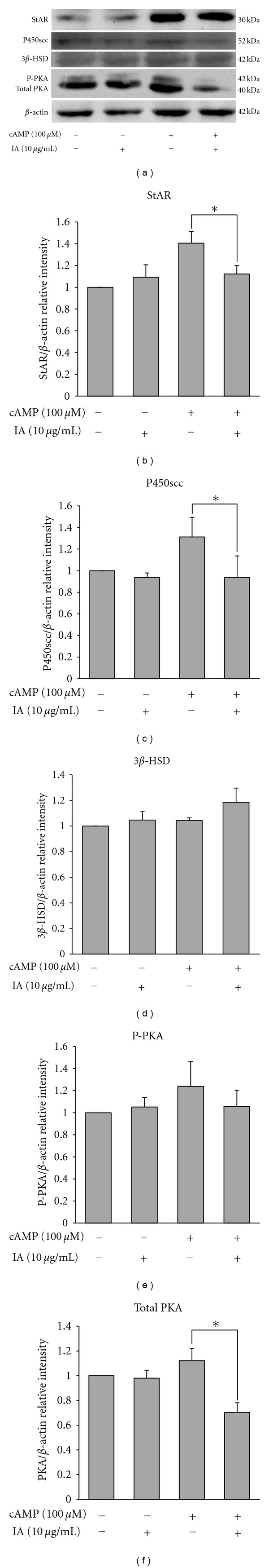
Effect of isocupressic acid on levels of steroidogenic proteins in MA-10 cells. MA-10 cells were treated with or without cAMP and IA for 4 h. The levels of steroidogenic proteins StAR, P450scc, and 3*β*-HSD were determined by Western blot analysis. Phosphorylation of PKA was also evaluated. Representative result of three independent experiments is shown in (a). Quantification of each blot is shown as multiples to control in (b, c, d, e, and f). Results are expressed as mean ± SEM. The statistically significant difference between two indicated groups is labeled (*P < 0.05).

Western blot analysis was used to further evaluate the effect of IA on proteins involved in steroidogenesis (i.e., StAR, P450scc, and 3*β*-HSD) and cAMP signaling (i.e., PKA and phospo-PKA). Representative results from three individual experiments are shown in Figure 7. After treatment with cAMP for 4 hours, cAMP did stimulate expression levels of StAR, P450scc, and PKA. After cotreatment with IA, the expression level of StAR, P450scc, and PKA was decreased. Although the phosphorylation of PKA was appearly stimulated by cAMP and decreased after cotreatment with IA, these results are not shown significant difference. 

## 4. Discussion

In the West, abortion in cattle induced by consumption of ponderosa pine needles is a serious problem in foothill and mountain rangelands [19]. Pine needle abortion often occurs during the late winter or early spring when cattle are in their third trimester of pregnancy. Moreover, late winter storms often force animals into pine tree stands for shelter, which increases their consumption of pine needles and exacerbates the abortion problem [20]. IA has been identified as the major component of pine needles responsible for its abortifacient effect [3, 5, 7]. After absorbed from the rumen, IA is metabolized to agathic acid by the liver and to dihydroagathic acid or tetra hydroagathic acid by the rumen [2]. Besides IA itself, its metabolite agathic acid has been recently shown to induce abortion in cattle [8].

IA was initially thought to act by decreasing placental perfusion; however, it did not appear to act directly on the uterine artery smooth muscle. Further, in 2004, a study demonstrated that IA did not affect bovine oocyte maturation or preimplantation embryo development [9]. Using a frozen-thawed bovine luteal cell system [21], we previously showed that IA decreased progesterone secretion from bovine luteal cells, suggesting that IA induces abortion by inhibiting luteal function. In this study, our purpose is to investigate the mechanism of how IA decreasing steroidogenesis ability, and our results indicated that the abortifacient actions may involve a post-cAMP mechanism.

Delivery of cholesterol to the mitochondrial inner membrane is a rate-limiting step of steroidogenesis [12]. Cholesterol import is a complex process involving interaction between StAR protein, which was previously purified and sequenced [22], and PBR [23, 24]. StAR protein, which is upregulated by trophic hormones like LH, is synthesized as a 37-kDa precursor protein that requires processing to a 30-kDa mature form to become functionally active [23]. StAR appears to be the initiator of cholesterol transport, whereas PBR functions as a gate for cholesterol entry into the mitochondria [25]. In the mitochondrial inner membrane, the rapid conversion of cholesterol into pregnenolone, which is a rate-limiting step of steroidogenesis, is catalyzed by P450scc [26]. Pregnenolone diffuses to the smooth endoplasmic reticulum where it is converted to progesterone by 3*β*-HSD. Progesterone is then metabolized to androstenedione by P450c17 and converted to testosterone by 17*β*-HSD [26]. However, P450c17 mRNA and enzyme activity cannot be detected in MA-10 cells; the main steroid produced by MA-10 cells is progesterone [27], which is also the main steroid produced by the corpus luteum. Therefore, in luteal cells and MA-10 cells, disrupting the function or expression of StAR, P450scc, or 3*β*-HSD may decrease progesterone production.

In the present study, we found that cAMP stimulates testosterone production in primary mouse Leydig cells, which is consistent with results of previous studies [28–30]. However, cotreatment with IA (0.5, 1, or 4 h) attenuated cAMP-stimulated testosterone production (Figure 2), consistent with results obtained with primary bovine luteum cells, suggesting that IA rapidly inhibits steroidogenesis in murine Leydig cells.

To characterize the molecular mechanisms underlying IA inhibition of steroidogenesis, we used a murine Leydig tumor cell line (MA-10) that increases progesterone production when stimulated with cAMP [31, 32]. We found that IA significantly decreased cAMP-stimulated progesterone production without cytotoxic effects (Figure 4).

Next, we evaluated the enzyme activities of P450scc, which converts cholesterol to pregnenolone, and 3*β*-HSD, which converts pregnenolone to progesterone. Incubation with pregnenolone markedly increases progesterone production by MA-10 cells [33]. We found that cotreatment with IA did not alter the effect of pregnenolone on progesterone production, indicating that IA does not regulate 3*β*-HSD activity (Figures 5(c) and 5(d)). To evaluate the role of P450scc in IA actions, we used a substrate of P450scc (22R-OHC), which freely crosses the aqueous space between the outer and inner mitochondrial membranes [34]. After confirming that incubation with 22R-OHC boosts progesterone production in MA-10 cells [33], we cotreated cells with IA and found that progesterone production was not decreased by IA treatment (Figures 5(a) and 3(b)). Since IA did not alter 3*β*-HSD activity, the lack of effect of IA on 22R-OHC-stimulated progesterone production shows that IA does not regulate P450scc activity. Taken together, our findings demonstrate that IA does not affect the activity of steroidogenic enzymes.

We next evaluated the effect of IA on the expression of steroidogenesis-associated genes PBR [25, 35], StAR, P450scc, and 3*β*-HSD by real-time PCR analysis as shown in Figure 6. Treatment with cAMP stimulated the expression of StAR, P450scc, and 3*β*-HSD mRNA. After cotreated with IA, P450scc mRNA was reduced while expression level of PBR and StAR mRNA was increased. To further investigate if IA functions on steroidogenic protein expression, western blot analysis was performed.

Treatment with cAMP for 4 h significantly stimulated expression of PKA. It is known that expression of StAR and P450scc are dependent on post-PKA pathway, mRNA, and protein expression of these two genes was stimulated by cAMP (Figures 6 and 7). After cotreatment with IA, expression of PKA was suppressed while phosphorylation of PKA was appearly suppressed. It was reasonable that expression of P450scc mRNA and protein stimulated with cAMP was suppressed by IA. Interesting, the protein expression of StAR was also suppressed by IA under stimulated with cAMP while the mRNA expression was not. Since the expression of StAR and P450scc is regulated by PKA, it was hard to figure out that why the protein expression of StAR was suppressed by IA under cAMP stimulated while the mRNA expression was not. Our results suggested that while steroidogenesis was suppressed by IA, some novel mechanisms may trigger the mRNA expression of StAR and PBR to facilitate the import of cholesterol into mitochondrion. However, the expression of StAR protein was suppressed by IA, steroids production was still suppressed.

**Figure 8:**
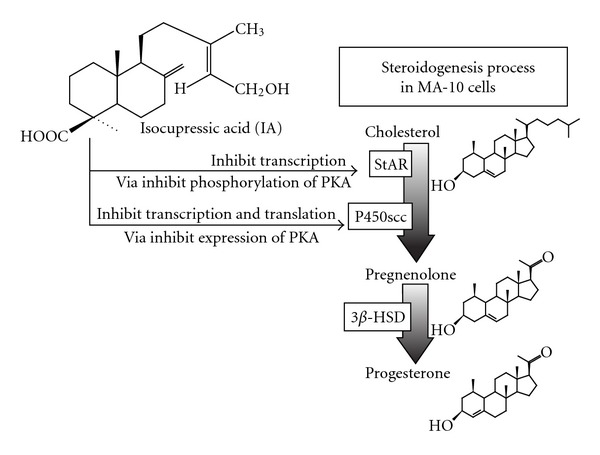
Possible pathway of how IA suppresses steroidogenesis of MA-10 cells.

 Taken together, our results suggest that IA appears to disrupt the post-cAMP signal pathway, inhibiting PKA expression and maybe phosphorylation, thereby reducing transcription and translation of P450scc and translation of StAR, then the progesterone production of MA-10 cells (Figure 8).

## 5. Conclusion

This study reports the molecular mechanisms underlying the inhibition of steroidogenesis by IA. We conclude that IA suppresses cAMP-induced steroidogenesis in murine Leydig cells. Our results indicate that reduced progesterone production in MA-10 cells is not mediated through the inhibition of P450scc and 3*β*-HSD activity, but by inhibiting transcription of P450scc and translation of both StAR and P450scc. The downregulation of transcription is due to the reduced phosphorylation of PKA caused by IA. Additional studies are needed to determine which transcription factor is targeted by IA, causing the downregulation of steroidogenesis.

## Figures and Tables

**Table 1:** Primer pairs for semiquantitative real-time PCR.

Target gene	GenBank accession number	Primer sequence	Product size (bp)
StAR	NM_011485.4	CGTGAGCGTGCGCTGTACCA	95
		TGACACCACTCTGCTCCGGCA	
P450scc	NM_019779.3	ACCGAGATGCTGGCAGGAGGG	119
		CGGGCAGCCAGGACTTCAGC	
3*β*-HSD	NM_008293.3	CAGCCAGGGGCCTTCGAGAC	143
		GCTGGCATTAGGGCGGAGCC	
PBR	NM_009775.4	GCTGGCTTTTGCCACCGTGC	119
		TGGCTGGCAGGGCTGCATTC	
*β*-actin	NM_007393.3	CCACCCGCGAGCACAGCTTC	102
		CGTTGTCGACGACCAGCGCA	

3*β*-HSD: 3*β*-hydroxysteroid dehydrogenase isoform: P450scc, cytochrome P450 cholesterol side-chain cleavage: PBR, peripheral-type benzodiazepine receptor: StAR, steroidogenic acute regulatory protein.
